# Perfil das alterações vasculares periféricas em dependentes de crack acompanhados em Centro de Atenção Psicossocial para Álcool e Drogas (CAPS-AD)

**DOI:** 10.1590/1677-5449.000716

**Published:** 2016

**Authors:** Antônio Fagundes da Costa, Leonardo Rodrigo Baldaçara, Sílvio Alves da Silva, Ana Célia de Freitas Ramos Tavares, Ederson de Freitas Orsolin, Vinícius Barros Prehl, Fernando Hirohito Beltran Gondo, Hernani Lopes Santana

**Affiliations:** 1 Universidade Federal do Tocantins – UFT, Curso de Medicina, Palmas, TO, Brasil.; 2 Hospital Geral Público de Palmas – HGPP, Serviço de Cirurgia Vascular, Palmas, TO, Brasil.

**Keywords:** crack, doença arterial periférica, abuso de drogas

## Abstract

**Contexto:**

O consumo de crack é um dos grandes desafios em saúde pública, e o uso dessa droga tem efeitos diretos na saúde de seus usuários.

**Objetivos:**

Avaliar o perfil das alterações vasculares em pacientes com dependência de crack em Centro de Atenção Psicossocial para Álcool e Drogas (CAPS-AD) e observar os possíveis efeitos vasculares periféricos.

**Métodos:**

Trata-se de um estudo observacional, descritivo, de corte transversal. Os pacientes da amostra foram submetidos a um questionário objetivo para avaliar questões demográficas, padrão de uso da droga, coexistência de diabetes melito, hipertensão arterial ou tabagismo, exame físico e ecográfico. Os dados foram sumarizados e analisados estatisticamente com teste qui-quadrado ou teste exato de Fisher.

**Resultados:**

A média de idade da amostra foi de 33,29 (±7,15) anos, e 74% eram do gênero masculino. A média de idade de início de uso da droga foi de 23,4 (±7,78) anos, com tempo médio de uso de 9,58 (±5,64) anos. O consumo médio diário de pedras de crack foi de 21,45 (±8,32) pedras. A alteração de pulsos em membros inferiores foi mais frequente em mulheres. A prevalência do espessamento da parede arterial nos membros inferiores foi de 94,8%. O tempo de uso da droga apresentou associação estatística (p = 0,0096) com alteração do padrão de curva espectral das artérias dos membros inferiores.

**Conclusões:**

Há alterações vasculares periféricas em usuários de crack. O tempo de uso da droga exerceu um maior impacto nesse sistema, o que sugere associação entre o uso do crack e a diminuição de fluxo arterial.

## INTRODUÇÃO

Os consumidores de crack apresentam mais problemas sociais e de saúde do que os consumidores de cocaína intranasal^1^. Os usuários dessa droga tendem a procurar tratamento mais precocemente do que os usuários de cocaína, além de aliarem ao consumo do crack outras drogas, como álcool, tabaco e maconha^1^.

Dados de uma pesquisa longitudinal com 131 consumidores de crack em São Paulo mostraram que a taxa de mortalidade desse grupo é sete vezes maior que a taxa de mortalidade global da população no mesmo período, sendo a maioria dos óbitos por causas externas e entre homens com menos de 30 anos, solteiros e de baixa escolaridade^2^.

Existe relação entre o abuso do crack e diversas patologias. Com relação ao sistema nervoso central, há relatos de quadro de vasculite que resultaram em infartos cerebrais, edema extenso e hemorragias cerebrais^3^. Quanto ao aparelho cardiovascular, foram relatados infarto agudo do miocárdio, cardiomiopatias, arritmias, endocardites, rotura de aneurismas, dissecção aórtica e trombose venosa^4^^-^ 8.

Quanto ao sistema vascular periférico, essa relação entre o uso do crack e a doença vascular periférica ainda não é totalmente conhecida. Apesar disso, é possível a ocorrência de trombose arterial por cocaína, que, apesar de ser pouco frequente, pode acometer vasos de pequeno e médio calibre, como as artérias cerebrais e coronarianas^9^. Nesse contexto, é possível que o mesmo processo ocorra nas artérias periféricas distais. Por sua vez, há relato de vasculopatia por vasoespasmo relacionado ao uso crônico de crack, que resultou em gangrena dos membros superiores e inferiores, com necessidade de amputações digitais e transfemoral^10^.

A doença arterial obstrutiva periférica (DAOP) é caracterizada por uma redução do fluxo sanguíneo para os membros, devido a processo oclusivo dos leitos arteriais^11^. Na maioria das vezes, sua causa é oriunda do processo aterosclerótico, mas pode ter outras etiologias, como arterites, espasmos, aneurismas ou tromboembolismo^12^. Os sintomas mais comuns dessa desordem são: ausência de pulsos distais e dor em membros superiores ou inferiores, que pode ocorrer após atividade física ou mesmo em repouso, com ou sem lesão cutânea^13^.

Informações sobre o consumo de crack no Brasil ainda são insuficientes. Nos últimos vinte anos, houve aumento na produção do conhecimento científico abrangendo os aspectos ligados ao consumo dessa droga^14^. Para o sistema vascular periférico, não há, ainda, estudos que comprovem a relação entre o uso do crack e o desenvolvimento de alterações vasculares periféricas. Nesse sentido, o presente estudo tem o objetivo de avaliar a prevalência de doenças vasculares periféricas em dependentes de crack acompanhados em um Centro de Atenção Psicossocial para Álcool e Drogas (CAPS-AD) e verificar se há relação entre o uso dessa droga e alterações vasculares periféricas.

## MÉTODOS

Este trabalho é um estudo do tipo observacional, descritivo, de corte transversal, com análise quantitativa dos dados. O estudo foi aprovado pelo Comitê de Ética e Pesquisa.

A população estudada foi composta por usuários de crack que estavam em tratamento em uma unidade do CAPS-AD entre março e agosto de 2015. A amostra tinha 58 pacientes. Os critérios de inclusão dos indivíduos na pesquisa foram: ter idade maior que 18 anos, ter sido diagnosticado como dependente químico do crack, estar em acompanhamento pela equipe multiprofissional do CAPS-AD, ter capacidade cognitiva e comportamental preservada no momento da entrevista, e apresentar ausência de diagnóstico firmado de vasculopatia prévia ao uso do crack. Os critérios de exclusão foram: ter idade menor que 18 anos, haver dúvida no diagnóstico de dependência química do crack, possuir déficit cognitivo ou comportamental no momento da entrevista, ou ter diagnóstico de vasculopatia prévia.

Para a coleta de dados, foi aplicado um questionário de múltipla escolha e realizado exame físico e ecográfico vascular com Doppler colorido dos membros superiores e inferiores nos pacientes. O formulário, desenvolvido pelos autores, abrangia aspectos epidemiológicos, padrão de consumo do crack, histórico clínico, exame físico e avaliação ecográfica vascular com Doppler colorido. A coleta de dados ocorreu na unidade CAPS-AD, onde os pacientes eram convidados a participar da pesquisa e esclarecidos quanto ao objetivo e aos procedimentos. Após leitura e assinatura do Termo de Consentimento Livre e Esclarecido (TCLE), prosseguia-se com o preenchimento do questionário. Posteriormente, os pacientes eram encaminhados para um dos consultórios do CAPS-AD, para que fosse realizado o exame físico e ecográfico.

O questionário levou em consideração os seguintes parâmetros demográficos: gênero (masculino e feminino), idade (dividida em faixas – entre 18 e 40 anos e entre 41 e 60 anos) e raça (caucasiano ou não caucasiano). Com relação à profissão, foi definido como parâmetro os indivíduos estarem empregados ou desempregados.

O padrão de consumo da droga levou em consideração a variável tempo de uso, que foi estratificada como ≤ 5 ou > 5 anos, e a variável quantidade de uso por dia, que foi dividida em ≤ 25 ou > 25 pedras fumadas por dia. Foi verificada também a frequência de uso semanal, dividida em duas faixas de uso: ≤ 3 ou > 3 dias/semana. Também houve questionamento sobre o uso ou não de outras drogas além do crack.

Foi pesquisado se os pacientes eram ou não portadores de diabetes melito e hipertensão arterial sistêmica, e se faziam uso ou não do tabaco e, quando positivo, o número de cigarros por dia. Durante a aplicação do questionário, foi perguntado a todos os pacientes sobre a presença ou não de dor em membros superiores ou inferiores e se havia ou não a presença de lesões cutâneas (tróficas).

As variáveis dependentes foram os pulsos arteriais, avaliados pelo exame físico, e o espessamento das paredes arteriais e o padrão de curva espectral, ambos avaliados pelo exame ecográfico vascular com Doppler. Os pulsos foram considerados “normais” quando palpados com facilidade e “alterados” quando palpados com dificuldade ou não palpados pelo exame físico, em pelo menos uma artéria distal dos membros superiores ou inferiores.

Para determinação dos parâmetros utilizados na avaliação ecográfica vascular com Doppler no presente estudo, foi realizada calibragem do equipamento por dois pesquisadores com experiência em ecografia vascular. A espessura da parede arterial foi considerada “alterada ou espessada” quando a parede arterial foi visualizada com nitidez ao corte ecográfico transverso e “normal” quando não visualizada. Quanto à avaliação da curva espectral para os membros superiores e inferiores, foi estabelecido como “normal” o padrão trifásico e “alterado” os padrões bifásicos e monofásicos. Tanto o espessamento quanto a curva espectral foram considerados alterados quando pelo menos uma artéria dos membros superiores ou inferiores apresentou alteração.

Os dados obtidos ao final da coleta foram sumarizados em planilha Excel e posteriormente processados pelo programa Epi Info™7. Para os cálculos estatísticos das variáveis categóricas, utilizou-se o teste qui-quadrado ou o teste exato de Fisher, quando indicado. O nível de significância estatística adotado foi de 0,05 (p).

## RESULTADOS

Na amostra de 58 indivíduos estudados, a média de idade foi de 33,29 (±7,15) anos. Com relação ao gênero, 43 (74,1%) foram do gênero masculino e 15 (25,9%) do gênero feminino. Dos pacientes avaliados, 14 (26,4%) eram caucasianos e 39 (73,5%) não caucasianos. Destes, 13 se disseram negros, 25 relataram ser pardos e 1 se declarou indígena. Com relação à profissão, 38 (65,5%) indivíduos afirmaram ter profissão e estar em frequente atividade de trabalho, enquanto 20 (34,5%) declararam estar desempregados.

Quando questionados sobre a idade de início de consumo do crack, a média foi de 23,4 (±7,78) anos, sendo que um paciente relatou ter iniciado o uso do crack com 11 anos. Na população estudada, o tempo médio de uso do crack foi de 9,58 (±5,64) anos, sendo que o menor tempo de uso da amostra foi de 1 ano e o maior, 26 anos. No estudo, foi avaliada a quantidade de consumo em pedras, sendo relatado um consumo diário médio de 21,45 (± 8,32) pedras. Sobre a frequência semanal de uso, a média avaliada foi de 5,74 (±1,81) dias, sendo que a maioria dos pacientes relatou uso diário ininterrupto, ou seja, 7 dias por semana. O uso de outras drogas foi encontrado em 45 (77,5%) pacientes, e 13 (22,5%) usavam exclusivamente o crack.

Questionados sobre diabetes melito, nenhum paciente se declarou diabético. Com relação a hipertensão arterial, 6 (10,3%) pacientes se disseram hipertensos, enquanto 52 (89,7%) eram normotensos. O tabagismo também foi pesquisado: 41 (70,7%) eram tabagistas crônicos e 17 (29,3%) se disseram não tabagistas frequentes. O consumo médio entre a população tabagista foi de 18,77 (±10,34) cigarros por dia. Com relação às variáveis dependentes, 21 (36,2%) indivíduos apresentaram dor em membros superiores, enquanto 37 (63,8%) não apresentaram.

Quanto a presença de lesões tróficas nos membros superiores, dois (3,44%) pacientes apresentaram ao menos uma lesão no curso do estudo, sendo que um deles apresentou também lesão em membro inferior. Nos membros inferiores, foram encontradas lesões tróficas também em dois (3,44%) pacientes.

Quanto a dor nos membros inferiores, 32 (55,17%) pacientes referiram dor esporádica à deambulação, enquanto 26 (44,82%) não referiram o sintoma. Nos membros superiores, o sintoma de dor foi referido por 21 (36,2%) pacientes, contra 37 (63,8%) que não mencionaram o sintoma.

Os pulsos arteriais nos membros superiores estavam normais em 54 (93,1%) pacientes, enquanto quatro (6,89%) apresentavam pulsos diminuídos. Já nos membros inferiores, em 25 (43,1%) pacientes os pulsos estavam normais, enquanto que em 33 (56,89%) estavam diminuídos, conforme a Tabela 1. Houve espessamento das artérias dos membros superiores em 37 (63,79%) pacientes, enquanto nos membros inferiores, o espessamento foi encontrado em 55 (94,82%) indivíduos, como demonstrado na Tabela 2.

**Tabela 1:** Distribuição da variável pulso arterial no exame clínico de acordo com valores normais e alterados conforme cada variável demográfica.

	**Pulsos MMSS**		**X** **^2^**	**p**	**Pulsos MMII**		**X** **^2^**	**p**
	**Normal**	**Alterada**			**Normal**	**Alterada**		
Gênero								
Masculino	42 (97,7%)	1 (2,3%)	5,4103	0,0493*	23 (53,5%)	20 (46,5%)	5,0426	0,0350**
Feminino	12 (80,0%)	3 (20,0%)			3 (20,0%)	12 (80,0%)		
Idade								
20-40	45 (91,8%)	4 (8,2%)	0,7891	1,0	22 (44,9%)	27 (55,1%)	0,0006	1,0
41-60	9 (100%)	0 (0,0%)			4 (44,4%)	5 (55,6%)		
**Etnia**								
Não caucasiano	36 (92,3%)	3 (7,7%)	0,0045	1,0	17 (43,6%)	22 (56,4%)	0,0023	1,0
Caucasiano	13 (92,9%)	1 (7,1%)			6 (42,9%)	8 (74,1%)		
Profissão								
Desempregado	16 (80,0%)	4 (20,0%)	8,1630	0,0114	6 (30,0%)	14 (70,0%)	2,7136	0,1642
Empregado	38 (100%)	0 (0,0%)			20 (52,6%)	18 (47,4%)		
Tempo de uso								
≤ 5 anos	15 (93,8%)	1 (6,2%)	0,144	1,0	7 (43,8%)	9 (56,2%)	0,0104	1,0
> 5 anos	39 (92,9%)	3 (7,1%)			19 (45,2%)	23 (54,8%)		
Quantidade								
≤ 25 pedras	28 (93,3%)	2 (6,7%)	0,0051	1,0	13 (43,3%)	17 (56,7%)	0,0561	1,0
> 25 pedras	26 (92,9%)	2 (7,1%)			13 (46,4%)	15 (53,6%)		
Frequência								
≤ 3 dias	13 (100%)	0 (0,0%)	1,2412	0,5652	7 (53,9%)	6 (46,2%)	0,5510	0,5351
> 3 dias	41 (91,1%)	4 (8,9%)			19 (42,2%)	26 (57,8%)		
Outras drogas								
Não	11 (84,6%)	2 (15,4%)	1,8801	0,2142	7 (53,9%)	6 (46,1%)	0,5510	0,5351
Sim	43 (95,6%)	2 (4,4%)			19 (42,2%)	26 (57,8%)		
Diabetes								
Não	54 (93,1%)	4 (6,9%)	-	-	26 (44,8%)	32 (55,2%)	-	-
Sim	0 (0,0%)	0 (0,0%)			0 (0,0%)	0 (0,0%)		
Hipertensão								
Não	48 (92,3%)	4 (7,7%)	0,4957	1,0	22 (42,3%)	30 (57,7%)	1,2906	0,3926
Sim	6 (100%)	0 (0,0%)			4 (66,7%)	2 (33,3%)		
**Tabagismo**								
Não	16 (94,1%)	1 (5,9%)	0,4778	1,0	9 (52,9%)	8 (47,0%)	0,8114	0,4236
Sim	38 (92,7%)	3 (7,3%)			17 (41,5%)	24 (58,5%)		

* *Odds ratio*: 10,50 - CI (0,99-110,36);

** *Odds ratio*: 4,60 - CI (1,13-18,65). MMSS: membros superiores; MMII: membros inferiores.

**Tabela 2:** Distribuição da variável espessamento arterial na ecografia de acordo com valores normais e alterados conforme cada variável demográfica.

	**Espessamento MMSS**		**X** ^2^	**p**	**Espessamento MMII**		**X^2^**	**p**
	**Normal**	**Alterada**			**Normal**	**Alterada**		
Gênero								
Masculino	17 (39,5%)	26 (60,5%)	0,7973	0,5348	1 (2,3%)	42 (97,7%)	2,7473	0,1610
Feminino	4 (26,7%)	11 (73,3%)			2 (13,3%)	13 (86,7%)		
Idade								
20-40	20 (40,8%)	29 (59,2%)	2,9048	0,1354	3 (6,1%)	46 (93,9%)	0,5811	1,0
41-60	1 (11,1%)	8 (88,9%)			0 (0%)	9 (100%)		
Etnia								
Não caucasiano	12 (30,8%)	27 (69,2%)	0,1156	0,7483	2 (5,1%)	37 (94,9%)	0,0783	1,0
Caucasiano	5 (35,7%)	9 (64,3%)			1 (7,1%)	13 (92,9%)		
Profissão								
Desempregado	6 (30,0%)	14 (70,0%)	0,5092	0,5716	2 (10,0%)	18 (90,0%)	1,4505	0,2709
Empregado	15 (39,5%)	23 (60,5%)			1 (2,6%)	37 (97,4%)		
Tempo de uso								
≤ 5 anos	6 (37,5%)	10 (62,5%)	0,0160	1,0	0 (0%)	16 (100%)	1,2052	0,5535
> 5 anos	15 (35,7%)	27 (64,3%)			3 (7,21%)	39 (92,9%)		
Quantidade								
≤ 25 pedras	11 (36,7%)	19 (63,3%)	0,0057	1,0	1 (3,3%)	29 (96,7%)	0,4285	0,6052
> 25 pedras	10 (35,7%)	18 (64,3%)			2 (7,1%)	26 (92,9%)		
Frequência								
≤ 3 dias	5 (38,5%)	8 (61,5%)	0,0369	1,0	1 (7,7%)	12 (92,3%)	0,2169	0,5401
> 3 dias	16 (35,6%)	29 (64,4%)			2 (4,4%)	43 (95,6%)		
Outras drogas								
Não	3 (23,1%)	10 (76,9%)	1,2506	0,3380	0 (0,0%)	13 (100%)	0,9139	1,0
Sim	18 (40,0%)	27 (60,0%)			3 (6,7%)	42 (93,3%)		
Diabetes								
Não	21 (36,2%)	37 (63,8%)	-	-	3 (5,2%)	55 (94,8%)	-	-
Sim	0 (0,0%)	0 (0,0%)			0 (0,0%)	0 (0,0%)		
Hipertensão								
Não	20 (38,5%)	32 (61,5%)	1,1063	0,4020	3 (5,8%)	49 (94,2%)	0,3650	1,0
Sim	1 (16,7%)	5 (83,3%)			0 (0,0%)	6 (100%)		
**Tabagismo**								
Não	5 (29,4%)	12 (70,6%)	0,4808	0,5601	1 (5,9%)	16 (94,1%)	0,0247	1,0
Sim	16 (39,0%)	25 (61,0%)			2 (4,9%)	39 (95,1%)		

MMSS: membros superiores; MMII: membros inferiores .

Na avaliação das curvas espectrais do exame ecográfico com Doppler colorido tanto dos membros superiores quanto dos inferiores, 49 (84,48%) pacientes apresentaram curva espectral alterada, enquanto 9 (15,51%) possuíam curvas normais, conforme apresentado na Tabela 3.

**Tabela 3:** Distribuição da variável curva espectral arterial na ecografia de acordo com valores normais e alterados conforme cada variável demográfica.

	**Curva MMSS**		**X^2^**	**p**	**Curva MMII**		**X^2^**	**p**
	**Normal**	**Alterada**			**Normal**	**Alterada**		
Gênero								
Masculino	9 (20,9%)	34 (79,1%)	3,7162	0,0941	8 (18,6%)	35 (81,4%)	1,2090	0,4217
Feminino	0 (0,0%)	15 (100%)			1 (6,7%)	14 (93,3%)		
Idade								
20-40	8 (16,3%)	41 (83,7%)	0,1578	1,0	8 (16,3%)	41 (83,7%)	0,1578	1,0
41-60	1 (11,1%)	8 (88,9%)			1 (11,1%)	8 (88,9%)		
**Etnia**								
Não caucasiano	5 (12,8%)	34 (87,2%)	0,0193	1,0	5 (12,8%)	34 (87,2%)	0,0193	1,0
Caucasiano	2 (14,3%)	12 (85,7%)			2 (14,3%)	12 (85,7%)		
Profissão								
Desempregado	0 (0,0%)	20 (100%)	5,6069	0,0208	0 (0,0%)	20 (100%)	5,6069	0,0208
Empregado	9 (23,7%)	29 (76,3%)			9 (23,7%)	29 (76,3%)		
Tempo de uso								
≤ 5 anos	2 (12,5%)	14 (87,5%)	0,1534	1,0	6 (37,5%)	10 (62,5%)	8,1448	0,0096*
> 5 anos	7 (16,7%)	35 (83,3%)			3 (7,1%)	39 (92,9%)		
Quantidade								
≤ 25 pedras	5 (16,7%)	25 (83,3%)	0,0626	1,0	5 (16,7%)	25 (83,3%)	0,0626	1,0
> 25 pedras	4 (14,3%)	24 (85,7%)			4 (14,3%)	24 (85,7%)		
Frequência								
≤ 3 dias	2 (15,4%)	11 (84,6%)	0,0002	1,0	3 (23,1%)	10 (76,9%)	0,7304	0,4044
> 3 dias	7 (15,6%)	38 (84,4%)			6 (13,3%)	39 (86,7%)		
Outras drogas								
Não	2 (15,4%)	11 (84,6%)	0,0002	1,0	4 (30,8%)	9 (69,2%)	2,9732	0,1024
Sim	7 (15,6%)	38 (84,4%)			5 (11,1%)	40 (88,9%)		
Diabetes								
Não	9 (15,5%)	49 (84,5%)	-	-	9 (15,5%)	49 (84,5%)	-	-
Sim	0 (0,0%)	0 (0,0%)			0 (0,0%)	0 (0,0%)		
Hipertensão								
Não	8 (15,4%)	44 (84,6%)	0,0067	1,0	6 (11,5%)	46 (88,5%)	6,0701	0,0420**
Sim	1 (16,7%)	5 (83,3%)			3 (50,0%)	3 (50,0%)		
**Tabagismo**								
Não	3 (17,7%)	14 (82,3%)	0,0832	1,0	4 (23,5%)	13 (76,47%)	1,776	0,4257
Sim	6 (14,6%)	35 (85,4%)			5 (12,2%)	36 (87,8%)		

* *Odds ratio*: 7,80 - CI (1,65-36,79);

** *Odds ratio*: 0,13 - CI (0,02-0,79). MMSS: membros superiores; MMII: membros inferiores.

## DISCUSSÃO

O presente trabalho é um dos primeiros estudos a se preocupar em investigar a associação entre o uso crônico do crack e alterações no sistema vascular periférico, sejam elas relatadas pelos pacientes, sejam detectadas por exame físico ou por exame complementar por imagem.

A avaliação dos pulsos arteriais periféricos é fundamental para o diagnóstico da doença arterial obstrutiva periférica^15^. Esses pulsos podem estar ausentes, diminuídos ou normais^16^. Neste estudo, a avaliação dos pulsos arteriais distais nos membros superiores evidenciou alteração em 6,89% da amostra, enquanto nos membros inferiores o percentual foi de 55,1%. De acordo com a literatura levantada, a prevalência da doença arterial obstrutiva periférica dos membros superiores varia entre 16 e 32%^17^^,^ 18.

Com relação à alteração de pulsos arteriais periféricos, fator preditor de doença vascular periférica, a variável demográfica que apresentou maior associação com a alteração dos pulsos foi o gênero. Neste estudo, 80% das mulheres demonstraram diminuição dos pulsos distais nos membros inferiores, enquanto 46,5% do gênero masculino apresentaram essa alteração. Panico (2009) descreveu prevalência de 3,5% de doença arterial periférica em mulheres e 9,8% em homens entre 30 e 54 anos^19^.

As variáveis relacionadas ao padrão de consumo do crack, como o tempo de uso, a quantidade de pedras e a frequência de uso, não apresentaram, individualmente, associação estatística com a alteração dos pulsos arteriais periféricos tanto superiores quanto inferiores.

A hipertensão e o tabagismo apresentam associação com a doença vascular periférica. A hipertensão está associada com todas as formas de doença cardiovascular. O tabagismo é apontado como responsável pelo adiantamento do diagnóstico da doença arterial periférica em uma década quando comparados fumantes e não fumantes^16^^,^ 20. Em nossa amostra, constituída predominantemente por indivíduos jovens, não houve variações significativas na percepção dos pulsos dos pacientes hipertensos ou tabagistas quando comparados com indivíduos não fumantes ou hipertensos.

O espessamento das paredes das artérias periféricas dos membros superiores foi encontrado em 63,7% dos pacientes, enquanto nos membros inferiores o espessamento foi encontrado em 94,8% dos pacientes. Apesar de nenhum paciente ter apresentado isquemia crítica, o espessamento das paredes arteriais periféricas dos membros inferiores pode estar relacionado com quadro de arterite em função do uso de crack^10^. Entende-se por isquemia crítica a ausência de pulsos associada a claudicação, dor em repouso ou lesão trófica. Balbir-Gurman (2001) relatou o caso de um homem jovem que apresentou hiperplasia da musculatura lisa de pequenos vasos arteriais com necrose de dedos após fenômeno de Raynaud induzido pelo uso do crack^21^.

Neste estudo, não foi encontrada associação estatística significativa entre variáveis demográficas, padrão de uso da droga ou comorbidades. Entretanto, a prevalência do espessamento das artérias periféricas distais dos membros inferiores aponta para uma alteração ainda muito precoce, possivelmente relacionada ao uso da droga, mas que ainda não captada pela percepção sintomática do paciente ou pelo exame físico da equipe médica.

A avaliação da curva espectral pelo exame ecográfico é uma importante ferramenta para avaliação do padrão de fluxo vascular. Dos 58 pacientes estudados, 84,4% apresentaram alteração da curva espectral de pelo menos uma artéria distal dos membros inferiores. A variável de padrão de consumo da droga que mais se associou à alteração da curva espectral foi o tempo de uso. Dos pacientes que relataram uso maior que 5 anos, 39 (92,9%) apresentaram alteração do padrão da curva espectral em pelo menos uma artéria dos membros inferiores. Nesse contexto, foi realizada também análise estatística entre as variáveis curva espectral e pulsos arteriais periféricos. Nos membros superiores, não houve associação entre as duas variáveis (p > 0,05). Entretanto, nos membros inferiores, dos 32 pacientes que apresentaram alteração de pulsos, 31 (96,8%) apresentaram, também, alteração da curva espectral ecográfica (p = 0,009). Zhou et al. (2004) relataram cinco casos de oclusão arterial dos membros – dois casos em usuários de cocaína e três casos em usuários de crack, todos em membros inferiores^9^.

Um estudo relatou uma prevalência de 52% de hipertensão em pacientes com isquemia de membros inferiores^22^. Entretanto, em nossa amostra, que possuía média de idade de 33,29 (±7,15), apenas 3 (50%) do total de pacientes hipertensos apresentaram curva espectral alterada nos membros inferiores, enquanto 46 (88,5%) pacientes sem hipertensão apresentaram alteração em pelo menos uma artéria periférica dos membros inferiores (p = 0,0420). Especula-se que o uso de anti-hipertensivo possa ter exercido efeito protetor sobre o sistema vascular periférico nesses pacientes. As demais variáveis demográficas, como sexo, idade e raça, as variáveis de padrão de uso e o tabagismo não apresentaram associação estatística com o padrão da curva espectral.

Segundo avaliação estatística, os 20 indivíduos desempregados (100%) apresentaram curva espectral de membros inferiores alterada. Entretanto, não encontramos justificativa fisiopatológica para essa correlação.

## CONCLUSÃO

A alteração da curva espectral dos membros inferiores, quando relacionada com a variável tempo de uso do crack, apresentou importante correlação estatística, podendo vir a ser um parâmetro utilizado no acompanhamento dos pacientes usuários de crack. Nesse sentido, percebeu-se também uma importante associação entre diminuição de pulsos e alteração da curva espectral dos membros inferiores, o que corrobora uma possível tendência de diminuição do fluxo de sangue arterial para essa região na população usuária do crack.

A prevalência do espessamento da parede arterial nos membros inferiores nos usuários de crack foi significativa. Apesar de não ter havido correlação estatística entre as variáveis demográficas, padrão de uso e comorbidades, quase 95% da amostra apresentaram espessamento da parede arterial nos membros inferiores.

A hipertensão arterial pode ter exercido papel protetor sobre a alteração da curva espectral nos membros inferiores, e este fenômeno pode estar relacionado com o uso de alguma medicação anti-hipertensiva. Entretanto, essa afirmação é apenas uma hipótese que demanda esclarecimentos futuros.

Os estudos sobre os efeitos do crack no sistema vascular periférico são escassos, o que mostra a necessidade de dar maior atenção à população usuária da droga e sua relação com a doença. Muitos casos são graves, com risco iminente de morte ou mutilações. Assim, são necessários novos estudos para uma melhor compreensão dos efeitos do crack na doença vascular arterial periférica.
