# Supplementary material for: Perfil das alterações vasculares periféricas em dependentes de crack acompanhados em Centro de Atenção Psicossocial para Álcool e Drogas (CAPS-AD)
Source: J Vasc Bras. 2016 Apr-Jun;15(2):126–33. [Article in Portuguese] doi: 10.1590/1677-5449.000716 (PMC5829706; doi:10.1590/1677-5449.000716)
Supplement: Translation

# Translation: Profile of peripheral vascular changes in crack-cocaine addicts receiving treatment at a Psychosocial Care Center for Alcohol and Drugs

**Authors:** Antônio Fagundes da Costa, Leonardo Rodrigo Baldaçara, Sílvio Alves da Silva, Ana Célia de Freitas Ramos Tavares, Ederson de Freitas Orsolin, Vinícius Barros Prehl, Fernando Hirohito Beltran Gondo, Hernani Lopes Santana

**Affiliations:** 1 Universidade Federal do Tocantins – UFT, Curso de Medicina, Palmas, TO, Brazil.; 2 Hospital Geral Público de Palmas – HGPP, Serviço de Cirurgia Vascular, Palmas, TO, Brazil.

**Keywords:** crack, peripheral arterial disease, drug abuse

## Abstract

**Background:**

Consumption of crack is one of the major challenges in public health and taking this drug has direct effects on the health of those who use it.

**Objectives:**

To evaluate the profile of vascular abnormalities in patients receiving treatment for crack dependency at a Psychosocial Care Center for Alcohol and Drugs and to observe possible peripheral vascular effects.

**Methods:**

The study design is observational, descriptive and cross-sectional. An objective questionnaire was administered to the patients in the sample to collect data on demographic details; drug use profile; and concomitant diabetes mellitus, arterial hypertension and/or smoking; and physical and ultrasound examinations were conducted. Data were summarized and analyzed statistically with the chi-square test or Fisher’s exact test.

**Results:**

The mean age of the sample was 33.29 (±7.15) years, and 74% were male. Mean age at onset of drug use was 23.4 (±7.78) years and mean time since onset was 9.58 (±5.64) years. Mean consumption of crack rocks was 21.45 (±8.32) per day. The rate of abnormal lower limb pulses was higher among women. The prevalence of artery wall thickening in lower limbs was 94.8%. Time since starting to use crack exhibited a statistically significant association (p = 0.0096) with abnormalities in the spectral curve profiles of lower limb arteries.

**Conclusions:**

Crack users exhibit peripheral vascular disorders. Length of time since starting to use the drug had the greatest impact on this system, suggesting an association between crack use and reduced arterial flow.

## INTRODUCTION

People who consume crack exhibit more social problems and health problems than people who consume intranasal cocaine.^1^ Crack users tend to seek treatment earlier than cocaine users and they also combine crack consumption with other drugs, such as alcohol, tobacco and marijuana.^1^

Data from a longitudinal study in São Paulo, Brazil, of 131 people who consume crack show that the mortality rate within this group is seven times greater than the overall mortality rate of the general population during the same period and that the majority of deaths are from external causes and among single men less than 30 years old who have a low educational level.^2^

There are relationships between crack abuse and many different pathologies. In relation to the central nervous system, there are reports of vasculitis cases that have resulted in cerebral infarcts, extensive edema and cerebral hemorrhages.^3^ In relation to the cardiovascular system, there have been reports of acute myocardial infarction, cardiomyopathies, arrhythmia, endocarditis, ruptured aneurysms, aortic dissection and venous thrombosis.^4^^-^ 8

In relation to the peripheral vascular system, the relationship between crack and peripheral vascular disease is not yet entirely clear. However, it is possible for arterial thrombosis to be caused by cocaine and, although infrequent, it can affect small and medium caliber vessels, such as cerebral and coronary arteries.^9^ It is also possible that the same process can occur in distal peripheral arteries. In turn, there is a report of vascular disease secondary to vasospasm related to chronic crack use, which resulted in gangrene of upper and lower limbs requiring digital and above-knee amputations.^10^

Peripheral arterial occlusive disease (PAOD) is characterized by a reduction in blood flow to the limbs caused by an occlusive process involving the arterial beds.^11^ In the majority of cases it is the result of an atherosclerotic process, but it can also have other etiologies, such as arterites, spasms, aneurysms or thromboembolism.^12^ The most common symptoms of this disorder are absent distal pulses and pain in the upper or lower limbs, which can occur after physical activity or even at rest, with or without cutaneous lesions.^13^

There is a lack of data on crack consumption in Brazil although production of scientific knowledge related to consumption of this drug has increased over the last twenty years.^14^ However, with relation to the peripheral vascular system, there are still no studies demonstrating a relationship between crack use and development of peripheral vascular disorders. In view of this, the objectives of this study are to evaluate the prevalence of peripheral vascular diseases in people with crack dependency who are receiving treatment at a Psychosocial Care Center for Alcohol and Drugs (CAPS-AD) and determine whether there is a relationship between use of this drug and peripheral vascular disorders.

## METHODS

The study design is observational, descriptive and cross-sectional, with quantitative analysis of data. The study was approved by the Research Ethics Committee.

The study population comprised crack users who were being treated at a CAPS-AD unit between March and August of 2015. The sample contained 58 patients. The criteria for inclusion of patients in the study were as follows: age over 18 years, having been diagnosed as being chemically dependent on crack, being treated by the multidisciplinary team at the CAPS-AD, having cognitive and behavioral capacities preserved at the time of interview, and not having been diagnosed with vascular disease prior to becoming a crack user. Exclusion criteria were: age less than 18 years, doubts relating to diagnosis of chemical dependency on crack, cognitive or behavioral deficits at the time of interview, or diagnosis of vascular disease prior to using crack.

Data were collected by administration of a multiple-choice questionnaire and by vascular physical examination and color Doppler ultrasound examination of patients’ upper and lower limbs. The data collection form was developed by the authors and covered epidemiological data, crack consumption profile, clinical history, and findings of a physical examination and a vascular assessment with color Doppler ultrasound. Data were collected at the CAPS-AD center, where patients were invited to take part in the study and were informed about the objective and the procedures involved. The questionnaire was administered after they had read and signed a free and informed consent form. The patients were then sent to one of the CAPS-AD consulting rooms where the physical and ultrasound examinations were conducted.

The questionnaire took into consideration the following demographic parameters: sex (male or female), age (classified in bands –18 to 40, or 41 to 60 years) and race (Caucasian or not Caucasian). The variable work was defined as whether participants were employed or unemployed.

The questions on drug consumption profile covered the variables time since use started, stratified as ≤ 5 or > 5 years; quantity consumed per day, classified as ≤ 25 or > 25 rocks smoked per day; frequency of use per week, divided into two bands, ≤ 3 or > 3 days/week; and use or non-use of drugs other than crack.

The patients were also asked whether or not they had diabetes mellitus and/or systemic arterial hypertension and whether they smoked tobacco and, if the answer was positive, the number of cigarettes smoked per day. During administration of the questionnaire, all patients were also asked whether they had or did not have pain in upper or lower limbs and whether or not they had cutaneous (trophic) lesions.

Dependent variables were arterial pulses, tested during the physical examination, and thickening of artery walls and the spectral curve profile, both assessed during the vascular ultrasound examination with Doppler. Pulses were considered “normal” when easily palpable and “abnormal” when palpated with difficulty or not palpable on physical examination, in at least one distal artery in an upper or lower limb.

The parameters used in this study for the sonographic vascular examination with Doppler were determined by calibrating the equipment, conducted by two researchers with experience in vascular echography. Artery wall thickness was defined as “abnormal or thickened” when the artery wall could be clearly seen on the transverse ultrasound scan and as “normal” when it could not be seen. The spectral curves for upper and lower limbs were defined as “normal” if the pattern was triphasic and “abnormal” if biphasic or monophasic. For both thickening and spectral curve, the patient was considered to have a disorder if at least one artery in an upper or lower limb exhibited an abnormality.

After collection, data were summarized in an Excel spreadsheet and then processed using Epi Info™ 7. The statistical calculations used for categorical variables, were the chi-square test or Fisher’s exact test, when appropriate. The level of statistical significance adopted was 0.05 (p).

## RESULTS

The mean age of the 58 people in the sample was 33.29 (±7.15) years. With relation to sex distribution, 43 (74.1%) were male and 15 (25.9%) were female. Fourteen (26.4%) members of the sample were Caucasians and 39 (73.5%) were not Caucasians, of whom 13 self-reported as black, 25 as brown and one self-reported as indigenous. With relation to work, 38 (65.5%) of the sample reported having a job and being engaged in frequent employment activity, while 20 (34.5%) reported being unemployed.

Mean age of first crack consumption was 23.4 (±7.78) years, although one patient reported having started crack use at 11 years of age. Mean time consuming crack in this study population was 9.58 (±5.64) years, ranging from 1 year to 26 years. The study also evaluated crack consumption in number of rocks and the mean daily consumption reported was 21.45 (± 8.32) rocks. Mean weekly frequency of use was 5.74 (±1.81) days, and the majority of patients reported uninterrupted daily use, i.e. 7 days per week. Use of other drugs was reported by 45 (77.5%) patients and 13 (22.5%) reported exclusively using crack.

When asked about diabetes mellitus, none of the patients stated they were diabetic. With relation to arterial hypertension, 6 (10.3%) patients reported being hypertensive, while 52 (89.7%) were normotensive. Smoking was also investigated: 41 (70.7%) were chronic smokers and 17 (29.3%) said they were not frequent smokers. Mean consumption by the subset of smokers was 18.77 (±10.34) cigarettes per day. With relation to the dependent variables, 21 (36.2%) participants had upper limb pain, while 37 (63.8%) did not.

The results for trophic ulcers of the upper limbs showed that two (3.44%) patients had at least one ulcer during the course of the study and one of these also had a lesion on a lower limb. A total of two (3.44%) patients had lower limb trophic ulcers.

In response to questions about pain in the lower limbs, 32 (55.17%) patients reported sporadic pain when walking, while 26 (44.82%) did not report this symptom. With relation to upper limbs, 21 (36.2%) patients reported pain while 37 (63.8%) did not mention this symptom.

Arterial pulses were present and normal in the upper limbs of 54 (93.1%) patients, while four (6.89%) patients had weak pulses. Pulses in the lower limbs of 25 (43.1%) patients were normal while in 33 (56.89%) patients lower limb pulses were weak, as shown in Table 1. There was thickening of upper limb arteries in 37 (63.79%) patients, while thickening was detected in the lower limbs of 55 (94.82%) participants, as shown in Table 2.

**Table 1:** Distribution of the variable clinical examination findings of arterial pulses, classified as normal or abnormal, by demographic variables.

	**Upper limb pulses**		X**^2^**	**p**	**Lower limb pulses**		**X** **^2^**	**p**
	**Normal**	**Abnormal**			**Normal**	**Abnormal**		
Sex								
Male	42 (97.7%)	1 (2.3%)	5.4103	0.0493*	23 (53.5%)	20 (46.5%)	5.0426	0.0350**
Female	12 (80.0%)	3 (20.0%)			3 (20.0%)	12 (80.0%)		
Age								
20-40	45 (91.8%)	4 (8.2%)	0.7891	1.0	22 (44.9%)	27 (55.1%)	0.0006	1.0
41-60	9 (100%)	0 (0.0%)			4 (44.4%)	5 (55.6%)		
**Ethnicity**								
Not Caucasian	36 (92.3%)	3 (7.7%)	0.0045	1.0	17 (43.6%)	22 (56.4%)	0.0023	1.0
Caucasian	13 (92.9%)	1 (7.1%)			6 (42.9%)	8 (74.1%)		
Employment								
Unemployed	16 (80.0%)	4 (20.0%)	8.1630	0.0114	6 (30.0%)	14 (70.0%)	2.7136	0.1642
Working	38 (100%)	0 (0.0%)			20 (52.6%)	18 (47.4%)		
Time using crack								
≤ 5 years	15 (93.8%)	1 (6.2%)	0.144	1.0	7 (43.8%)	9 (56.2%)	0.0104	1.0
> 5 years	39 (92.9%)	3 (7.1%)			19 (45.2%)	23 (54.8%)		
Quantity								
≤ 25 rocks	28 (93.3%)	2 (6.7%)	0.0051	1.0	13 (43.3%)	17 (56.7%)	0.0561	1.0
> 25 rocks	26 (92.9%)	2 (7.1%)			13 (46.4%)	15 (53.6%)		
Frequency								
≤ 3 days per week	13 (100%)	0 (0.0%)	1.2412	0.5652	7 (53.9%)	6 (46.2%)	0.5510	0.5351
> 3 days per week	41 (91.1%)	4 (8.9%)			19 (42.2%)	26 (57.8%)		
Other drugs								
No	11 (84.6%)	2 (15.4%)	1.8801	0.2142	7 (53.9%)	6 (46.1%)	0.5510	0.5351
Yes	43 (95.6%)	2 (4.4%)			19 (42.2%)	26 (57.8%)		
Diabetes								
No	54 (93.1%)	4 (6.9%)	-	-	26 (44.8%)	32 (55.2%)	-	-
Yes	0 (0.0%)	0 (0.0%)			0 (0.0%)	0 (0.0%)		
Hypertension								
No	48 (92.3%)	4 (7.7%)	0.4957	1.0	22 (42.3%)	30 (57.7%)	1.2906	0.3926
Yes	6 (100%)	0 (0.0%)			4 (66.7%)	2 (33.3%)		
**Smoking**								
No	16 (94.1%)	1 (5.9%)	0.4778	1.0	9 (52.9%)	8 (47.0%)	0.8114	0.4236
Yes	38 (92.7%)	3 (7.3%)			17 (41.5%)	24 (58.5%)		

* Odds ratio: 10.50 - CI (0.99-110.36);

** Odds ratio: 4.60 - CI (1.13-18.65).

**Table 2:** Distribution of the variable arterial thickening in sonographic examination, classified as normal or abnormal, by demographic variables.

	**Upper limb thickening**		**X** **^2^**	**p**	**Lower limb thickening**		**X** **^2^**	**p**
	**Normal**	**Abnormal**			**Normal**	**Abnormal**		
Sex								
Male	17 (39.5%)	26 (60.5%)	0.7973	0.5348	1 (2.3%)	42 (97.7%)	2.7473	0.1610
Female	4 (26.7%)	11 (73.3%)			2 (13.3%)	13 (86.7%)		
Age								
20-40	20 (40.8%)	29 (59.2%)	2.9048	0.1354	3 (6.1%)	46 (93.9%)	0.5811	1.0
41-60	1 (11.1%)	8 (88.9%)			0 (0%)	9 (100%)		
Ethnicity								
Not Caucasian	12 (30.8%)	27 (69.2%)	0.1156	0.7483	2 (5.1%)	37 (94.9%)	0.0783	1.0
Caucasian	5 (35.7%)	9 (64.3%)			1 (7.1%)	13 (92.9%)		
Employment								
Unemployed	6 (30.0%)	14 (70.0%)	0.5092	0.5716	2 (10.0%)	18 (90.0%)	1.4505	0.2709
Working	15 (39.5%)	23 (60.5%)			1 (2.6%)	37 (97.4%)		
Time using crack								
≤ 5 years	6 (37.5%)	10 (62.5%)	0.0160	1.0	0 (0%)	16 (100%)	1.2052	0.5535
> 5 years	15 (35.7%)	27 (64.3%)			3 (7.21%)	39 (92.9%)		
Quantity								
≤ 25 rocks	11 (36.7%)	19 (63.3%)	0.0057	1.0	1 (3.3%)	29 (96.7%)	0.4285	0.6052
> 25 rocks	10 (35.7%)	18 (64.3%)			2 (7.1%)	26 (92.9%)		
Frequency								
≤ 3 days per week	5 (38.5%)	8 (61.5%)	0.0369	1.0	1 (7.7%)	12 (92.3%)	0.2169	0.5401
> 3 days per week	16 (35.6%)	29 (64.4%)			2 (4.4%)	43 (95.6%)		
Other drugs								
No	3 (23.1%)	10 (76.9%)	1.2506	0.3380	0 (0.0%)	13 (100%)	0.9139	1.0
Yes	18 (40.0%)	27 (60.0%)			3 (6.7%)	42 (93.3%)		
Diabetes								
No	21 (36.2%)	37 (63.8%)	-	-	3 (5.2%)	55 (94.8%)	-	-
Yes	0 (0.0%)	0 (0.0%)			0 (0.0%)	0 (0.0%)		
Hypertension								
No	20 (38.5%)	32 (61.5%)	1.1063	0.4020	3 (5.8%)	49 (94.2%)	0.3650	1.0
Yes	1 (16.7%)	5 (83.3%)			0 (0.0%)	6 (100%)		
**Smoking**								
No	5 (29.4%)	12 (70.6%)	0.4808	0.5601	1 (5.9%)	16 (94.1%)	0.0247	1.0
Yes	16 (39.0%)	25 (61.0%)			2 (4.9%)	39 (95.1%)		

Evaluation of the spectral curves from the color Doppler ultrasound examinations showed that for both upper and lower limbs 49 (84.48%) patients exhibited abnormal spectral curves, while 9 (15.51%) had normal curves, as shown in Table 3.

**Table 3:** Distribution of the variable arterial spectral curve in sonographic examination, classified as normal or abnormal, by demographic variables.

	**Upper limb curve**		**X** **^2^**	**p**	**Lower limb curve**		**X** **^2^**	**p**
	**Normal**	**Abnormal**			**Normal**	**Abnormal**		
Sex								
Male	9 (20.9%)	34 (79.1%)	3.7162	0.0941	8 (18.6%)	35 (81.4%)	1.2090	0.4217
Female	0 (0.0%)	15 (100%)			1 (6.7%)	14 (93.3%)		
Age								
20-40	8 (16.3%)	41 (83.7%)	0.1578	1.0	8 (16.3%)	41 (83.7%)	0.1578	1.0
41-60	1 (11.1%)	8 (88.9%)			1 (11.1%)	8 (88.9%)		
**Ethnicity**								
Not Caucasian	5 (12.8%)	34 (87.2%)	0.0193	1.0	5 (12.8%)	34 (87.2%)	0.0193	1.0
Caucasian	2 (14.3%)	12 (85.7%)			2 (14.3%)	12 (85.7%)		
Employment								
Unemployed	0 (0.0%)	20 (100%)	5.6069	0.0208	0 (0.0%)	20 (100%)	5.6069	0.0208
Working	9 (23.7%)	29 (76.3%)			9 (23.7%)	29 (76.3%)		
Time using crack								
≤ 5 years	2 (12.5%)	14 (87.5%)	0.1534	1.0	6 (37.5%)	10 (62.5%)	8.1448	0.0096*
> 5 years	7 (16.7%)	35 (83.3%)			3 (7.1%)	39 (92.9%)		
Quantity								
≤ 25 rocks	5 (16.7%)	25 (83.3%)	0.0626	1.0	5 (16.7%)	25 (83.3%)	0.0626	1.0
> 25 rocks	4 (14.3%)	24 (85.7%)			4 (14.3%)	24 (85.7%)		
Frequency								
≤ 3 days per week	2 (15.4%)	11 (84.6%)	0.0002	1.0	3 (23.1%)	10 (76.9%)	0.7304	0.4044
> 3 days per week	7 (15.6%)	38 (84.4%)			6 (13.3%)	39 (86.7%)		
Other drugs								
No	2 (15.4%)	11 (84.6%)	0.0002	1.0	4 (30.8%)	9 (69.2%)	2.9732	0.1024
Yes	7 (15.6%)	38 (84.4%)			5 (11.1%)	40 (88.9%)		
Diabetes								
No	9 (15.5%)	49 (84.5%)	-	-	9 (15.5%)	49 (84.5%)	-	-
Yes	0 (0.0%)	0 (0.0%)			0 (0.0%)	0 (0.0%)		
Hypertension								
No	8 (15.4%)	44 (84.6%)	0.0067	1.0	6 (11.5%)	46 (88.5%)	6.0701	0.0420**
Yes	1 (16.7%)	5 (83.3%)			3 (50.0%)	3 (50.0%)		
**Smoking**								
No	3 (17.7%)	14 (82.3%)	0.0832	1.0	4 (23.5%)	13 (76.47%)	1.776	0.4257
Yes	6 (14.6%)	35 (85.4%)			5 (12.2%)	36 (87.8%)		

* Odds ratio: 7.80 - CI (1.65-36.79);

** Odds ratio: 0.13 - CI (0.02-0.79).

## DISCUSSION

This is one of the first studies to investigate the association between chronic crack consumption and disorders of the peripheral vascular system, whether reported by the patients themselves or detected by physical examination or imaging exams.

Assessment of peripheral arterial pulses is an essential part of diagnosing peripheral arterial occlusive disease.^15^ Pulses may be absent, weak or normal.^16^ In this study, assessment of distal arterial pulses in the upper limbs found evidence of abnormalities in 6.89% of the sample, while the percentage in the lower limbs was 55.1%. According to the literature reviewed, the prevalence of peripheral arterial occlusive disease varies from 16 to 32% in the upper limbs.^17^^,^ 18

With relation to peripheral arterial pulses, which are a predictive factor for peripheral vascular disease, the demographic variable with the greatest association was sex. In this study, 80% of the women had reduced distal pulses in the lower limbs, while 46.5% of the men had this abnormality. Panico (2009) reported a 3.5% prevalence of peripheral arterial disease in women and 9.8% in men aged 30 to 54 years.^19^

Variables related to the participants’ profile of crack consumption, such as time since starting to take crack, number of rocks and frequency of use, did not individually exhibit a statistical association with abnormal peripheral arterial pulses, whether in the upper or lower limbs.

Hypertension and smoking are associated with peripheral vascular disease. Hypertension is associated with all forms of cardiovascular disease. Smoking has been identified as responsible for bring forward a diagnosis of peripheral arterial disease by a decade, when smokers and non-smokers are compared.^16^^,^ 20 In our sample, made up predominantly of young people, there were no significant variations in detection of the pulses of hypertense patients or smokers in comparison with participants who were not smokers or hypertense.

Thickening of the walls of peripheral arteries in the upper limbs was observed in 63.7% of the patients, while thickening in the lower limbs was observed in 94.8% of the patients. Although none of the patients had critical ischemia, thickening of the walls of peripheral arteries in the lower limbs may be related to arteritis secondary to crack use.^10^ Critical ischemia is defined as absent pulses combined with claudication, pain at rest or trophic ulcers. Balbir-Gurman (2001) reported a case of a young man who exhibited hyperplasia of the smooth musculature of small arterial vessels with necrosis of the fingers after the Raynaud phenomenon, induced by crack use.^21^

In this study, no statistically significant associations were found with demographic variables, drug use profile or comorbidities. However, the prevalence of thickening of distal peripheral arteries in the lower limbs indicates a disorder with very early onset, possibly related to drug consumption, but not yet perceived as symptomatic by the patient or captured by the medical team during physical examination.

Assessment of the spectral curve by ultrasonographic examination is an important tool for analyzing vascular flow patterns. Of the total sample of 58 patients studied, 84.4% exhibited abnormalities of the spectral curve in at least one distal artery in the lower limbs. The drug consumption profile variable that was most strongly associated with abnormal spectral curves was time since onset of use. Among patients who reported using crack for more than 5 years, 39 (92.9%) exhibited abnormalities of the spectral curve in at least one lower limb artery. In view of this, a statistical analysis was also conducted of the relationship between the variables spectral curve and peripheral arterial pulses. There was no association between the two variables for the upper limbs (p > 0.05). However, in the lower limbs, 31 of the 32 (96.8%) patients who exhibited abnormal pulses also exhibited altered ultrasonographic spectral curves (p = 0.009). Zhou et al. (2004) described five cases of occlusion of arteries in the limbs – two cases in cocaine users and three cases in crack users, all in lower limbs.^9^

One study reported a 52% prevalence of hypertension among patients with lower limb ischemia.^22^ However, in our sample, with a mean age of 33.29 (±7.15), only 3 (50%) of the hypertense patients exhibited an abnormal spectral curve in the lower limbs, while 46 (88.5%) patients without hypertension exhibited an abnormality in at least one peripheral artery of the lower limbs (p = 0.0420). It is postulated that use of antihypertensives may have had a protective effect on the peripheral vascular system in this sample of patients. None of the other demographic variables, such as sex, age and race, drug use profile variables, or smoking, exhibited statistical associations with the spectral curve data.

According to the statistical analysis, all 20 of the unemployed participants (100%) exhibited abnormal lower limb spectral curves. However, we did not identify any pathophysiological explanation for this correlation.

## CONCLUSIONS

Abnormalities of lower limb spectral curves exhibited a robust statistical correlation with the variable time since starting to use crack, and could come to be a parameter used for monitoring patients who are crack users. There was also an association between attenuated pulses and abnormal lower limb spectral curve, which supports the possibility of a tendency to reduced arterial blood flow to this area in the population of crack users.

There was a considerable prevalence of thickening of the walls of lower limb arteries was among these crack users. Although there was no statistical correlation with demographic variables, profile of drug use or comorbidities, almost 95% of the sample exhibited thickening of artery walls in the lower limbs.

Arterial hypertension may have played a protective role with relation to abnormal spectral curves in the lower limbs, and this phenomenon may be related to use of some type of antihypertensive medication. However, this statement is merely a hypothesis that will require further investigation.

There are few studies of the effects of crack on the peripheral vascular system, which shows that there is a need to pay greater attention to the drug user population and its relationship with disease. Many cases are severe, involving imminent risk of death or mutilation. Further studies are therefore needed to improve understanding of the effects of crack on vascular peripheral arterial diseases.
